# A Reply to Dr. Rhein’s Answer

**Published:** 1904-07

**Authors:** E. K. Wedelstaedt

**Affiliations:** St. Paul, Minn.


### A REPLY TO DR. RHEIN'S ANSWER.

BY E. K. WEDELSTAEDT, ST. PAUL, MINN.

I HAVE read the "Answer" of Dr. Rhein, which appeared in the March number of this journal, also his remarks, which appear in the *Dental Cosmos* for April.

As was stated in my discussion, when a man of the doctor's standing will make changes in his methods, there is hope for others. After comparing the terms which he uses in his essay with those he is quoted as using in the *Dental Cosmos,* I feel, if nothing is gained otherwise, that the educational value of the discussion of his ideas is productive of great good. Dr. Rhein is to be congratulated for placing to one side the use of ambiguous terms and using in their place definite terms. With this example before us, there is a chance that the use of such terms as "floor of the cavity," "putting in fillings in the mouth," "cavities between the teeth," "anterior," "posterior," "canine," etc., etc., will soon disappear from print and exact terms be used.

There is so much to be discussed, and so little time mine in which to formulate ideas, that I am compelled to write most hastily. Let the reader understand what is meant by the use of the two terms, cohesive or annealed gold, and soft or unannealed gold. By cohesive gold is meant gold which is annealed just prior to being placed in the cavity. By soft gold is meant gold which is not annealed prior to being placed in the cavity. What is printed on the book in which the gold comes from the dental depot makes no difference. In using these terms in this way, there cannot be any "soft cohesive" gold.

An examination of a section of a tooth which has had a cavity in the proximo-occlusal surface filled with gold, anchoring the gold in cement, which has been "plastered over the floor of the gingival seat" prior to placing the gold, will show a layer of cement between the tooth and the gold. Has not the doctor become somewhat mixed in the use of terms? It is a very difficult matter for me to believe the statement as it appears before me in print. If Dr. Rhein means what he says, then I have no time at my disposal to spend in discussing any such unscientific method.

It is alleged in the "Answer" that a certain gold filling condensed with the electric mallet had a specific gravity of 19.87.

The usual specific gravity which is accredited cast gold is 19.3. Higher specific gravities, such as 19.42 for hammered gold, have been obtained. A specific gravity of 19.3 for pure gold has, however, been accepted by all scientists. When, therefore, it is alleged that a specific gravity of 19.87 has been obtained, and that by using an electric mallet for the purpose of condensing the gold to a certain filling, all thinking men are compelled to ask, first, will the doctor please send the filling to another chemist and obtain a certificate from him regarding the specific gravity? Secondly, if he is unwilling to do this, will he not ask his chemist to make another specific gravity after first balancing his scales? It is an easy matter to obtain a specific gravity, but very often errors creep in.

Some time ago a man, who for twenty years had used the electric mallet, made some fillings for me in the steel cavity block, using a No. 3 Webb plugger in his mallet for condensing the gold. The gold used was Williams's No. 4 cohesive foil. A sheet of foil was divided into thirty-two equal pieces, and each piece rolled into a loose pellet. Each piece of gold was annealed just prior to being placed into the cavity. Not less than thirty-five seconds of malleting was given any of the pieces of gold, and so many pieces received seventy-five seconds that I was first inclined to adopt the last-named figure as the average. A number of specific gravities were made by two different persons, and but ten specific gravities could be obtained.

Some reference has been made to the exhibit of fillings which can be found on pages 749 and 750 of the *Dental Cosmos* for 1895. The operator who made fillings Nos. 41, 42, and 43 has used the electric mallet for twenty-two years, and he informs me that he knows how to use it also. I am conversant with the manner as well as the method used in making these fillings. Anybody who is interested in this subject can make a comparison of the different specific gravities in the exhibit and draw his own conclusions. It is not necessary for me to say anything regarding the differences existing between the different specific gravities. They tell their own story. I am, however, very sorry to say that there seems to be a very great difference between 10.7 and 19.87, or even between 13.2 and 19.87.

Others are now beginning to discuss our expressed views. They have said something about the use of cohesive and soft gold, as well as polished enamel margins. This is about the place that calls for some plain English. I do not care to give expression to thoughts which but few will believe, notwithstanding the fact that they may be but too true. But if the issue is forced upon me, it is an easy matter to say what is necessary. Altogether too many men become possessed with ideas, and too often it is imagined that these ideas are facts. Too often when we come to simmer down many of these ideas, we find that they are based upon the veriest suppositions. We have made many contentions regarding them, and when we find that they are worthless, how many men have the moral courage to go into print and acknowledge the error? Well, not very many. There are many men in our profession who imagine that they are making water-tight margins with cohesive gold against polished or even planed enamel margins. I do not care to enter into a lengthy discussion regarding any man's ability to do this, and I wish to say right here that unless I had been perfectly aware of what I was writing about I should not have said what I did about polished enamel margins and the use of adhesive gold. For the past thirteen years I have been making a careful study of this subject, and I have also spent much time in observing the methods employed by other operators while packing cohesive gold. This is neither the time nor the place to discuss the value of cohesive or soft gold, for every man of any knowledge in our profession knows how valuable both these materials are in their proper place. The quickest way for the reader to satisfy himself regarding the relative value of using cohesive and soft foils is to obtain two glass tubes, three millimetres in diameter. These glass tubes can be cemented into a block of wood in such a manner that the cavity in the glass tube will be about two and one-half or three millimetres deep. These two glass tubes can be filled, one with cohesive and the other with soft gold. After the fillings have been made, they can be removed from the wood, examined, and thereafter tested in aniline. An examination of the fillings prior to their being placed in aniline will tell a far better story of the relative value of the different golds used than any words of mine. In the aniline test it will be found that both fillings leak. If there is a desire on the part of the experimenter to test his ability for making water-tight margins with cohesive gold, let him grind the inside of one of the glass tubes with a coarse stone, fill it with cohesive gold, remove it from the block, and test it in aniline. A number of the members of the Black Club have made this experiment, and have made non-leakable fillings. No man has, however, ever succeeded in adapting cohesive gold against the polished surfaces of glass tubes and made a non-leakable filling. (See page 955, *Dental Cosmos,* 1891.) But cohesive gold has been packed against the roughened surfaces of glass tubes, and water-tight fillings have been made. I merely give the results of my own experiments, and leave it for the reader to draw his own conclusions regarding the ability of any man to pack cohesive gold against differently prepared enamel margins.

After making about so many experiments with gold, a man begins to have a certain respect for cohesive gold which he did not imagine it was possible for mortal to have. It may be an easy matter to handle cohesive gold, but I am free to confess that the more of it I use, the greater is my respect for it and the more dissatisfied I become with myself, my operations, and the gold. And in conversation with others who have also made laboratory experiments, studied filled extracted teeth, and who know much more than I shall ever know, I find that they give expression to similar opinions, which are based upon the results of experimental research, observation, and a study of the gold itself. Where a man says that it is an easy matter to make tight margins with cohesive gold, I am quickly made aware that that man has much more to learn than he knows of, for had he any knowledge of the action of cohesive gold under the plugger-point he would be more careful in his statements. Where men say that they can do this, that, and the other thing with cohesive gold, I merely wish to observe their methods while operating. Let me illustrate what I mean. Recently, while at a public clinic, a man came to me and said, "Come across the room with me, for I wish you to see the best operator in this country pack gold." I accompanied the young man as was requested. When we reached the operator's chair he had his back turned to us and was just placing a mat of gold to his partly completed operation. It was only necessary to observe the methods which he employed in condensing the piece of gold which he had just placed to his filling to prove that the man did not have the remotest idea of handling his gold. He displayed a total lack of knowledge regarding the important part of employing lines of force and principles of instrumentation. He was in total ignorance of any knowledge pertaining to these subjects. I

observed the man place his second mat of gold, and thought, here is a chance to do some real charity work. Before, however, I had a chance to say anything to him, he turned and said to me, "How do you like it? Is it not fine? There is no man in this country my equal when it comes to 'putting in' gold." I wisely held my peace; silence is often golden. Perhaps such might have been the case in this discussion, and yet the individual *per se* is of little importance, but the advance and progress of the men in the dental profession is all important. Whether it is possible for Dr. Rhein to have me look at his methods from his stand-point, or whether I be able to have him look at things from my point of view, makes little difference; but it is of greatest value to us all if some of the readers of these words become sufficiently interested to make the experiments to which attention has been called.

It makes very little difference to the seeker of truth what either Dr. Rhein or I say. Two men with such widely diverse views can never convince each other, so it remains then for the reader to take the best out of what has been written, make the experiments, and judge for himself. Discussions of any subject should be the means of assisting others to get a better hold on the truth, which should enable them to be of greater use to humanity by placing the truth in use. Personalities, ridiculing the expressed views of others, etc., are displays of weakness, and have no place in a discussion of scientific subjects. Readers of dental periodicals wish the salient features brought out, and in any discussion all that is necessary is an expression of opinion which should be the means of assisting others towards having a better understanding of the essentials. If the reader of these words will but make the experiments to which attention has been called, he can ascertain for himself all that is necessary.

There has been a discussion in the "Answer" of the electric mallet. If we believe in the theory that it is necessary to use certain lines of force in packing gold, then the electric mallet has but a limited use. (As cavities in the proximo-occlusal surfaces of molars and bicuspids have been dealt with, my discussion will be confined to dealing with the cavities spoken of.) The electric mallet can only be used for packing gold in cavities in the mesial surfaces, with here and there a cavity in the distal surface. The cavities are opened from the occlusal surface, and direct access can thus be obtained and correct lines of force used in packing the gold. Where the cavity is situated in the distal surface of molars, and more especially lower bicuspids, the electric mallet is absolutely useless, provided correct lines of force are to be used. Reverse instruments are, however, absolutely necessary if the operator desires to use correct lines of force, which must be used, provided water-tight fillings are to be made. Nor is this all. The condensing power of the electric mallet is so slight that it can readily be called a mere surface condenser. It has no value as a condensing instrument *per se.* To prove this assertion take a piece of smooth lead or a piece of smooth wood. With a No. 3 Webb plugger in the electric mallet, make a row of indentations across one end of the lead or board. Remove the plugger from the mallet and make a row of indentations by using the hand-mallet on the end of the plugger. Another row of indentations can be made by using hand-pressure. If a comparison with the power of an automatic mallet is desired, place the same sized plugger in it and make a row of indentations. Anybody making this experiment will be in a position to know whether or not there is any truth in my allegation that the electric mallet is merely fit for surface condensation. (See remarks made by Dr. Perry, on page 221, *Dental Cosmos,* 1878.) Those interested should make this experiment, for to comprehend we must first apprehend, and this apprehension leads to our having an understanding of the subject. After the experiment has been made, we can better comprehend the experiments of Dr. Black (see pages 741–746, *Dental Cosmos,* 1895), and are capable of better understanding the efforts which he displayed in obtaining a specific gravity of 19.42. If it was necessary for him to use the methods which he employed for this purpose to obtain any such result, how is it possible to condense gold with an electric mallet, that condenses the surface only, to obtain a specific gravity of 19.87? I could with equal truth say, I took four ounces of gold and refined it four times. Thereafter I took it to Dr. Lehnen, a well-known chemist of this city, who assured me that it was 27-carat fine. Should such a thing happen to me, I should at once question the sanity of Dr. Lehnen and in future consult with a different chemist. I should not publish Dr. Lehnen's statement and ask the men in the dental profession, "Am I too presumptuous in claiming that gold" of greater purity than 24-carat can be obtained?

Here are four facts with which all thinking dentists are conversant:

1. Where cohesive gold fillings have a specific gravity of 17 or 18 we all know what a difficult matter it is to cut into them for the purpose of anchoring a proximating filling. A bur will slip and slide and will not "bite." A drill must be used to enter the filling.

2. Every man with any experience knows that gold fillings condensed with the electric mallet are as easily entered with a bur as cutting so much putty. In his essay and in the answer to my discussion, Dr. Rhein speaks of using a bur on that portion of the filling previously placed and recommencing the packing of the gold on this freshened surface.

3. Since this "Answer" has been published every man I have asked has informed me that where he attempted to anchor one filling into another, he having made the original filling, he was compelled to start in a definite retention pit in the filling. I do not believe a union of the kind Dr. Rhein speaks of can be made if the gold is cohesive gold and of 18 specific gravity. Even if it could be made, the gold would be held together by interserration, and not molecule adhering to molecule.

4. It is a fact that gold fillings condensed with the electric mallet are always so odoriferous. Remove one of these fillings from the teeth, roll it between the thumb and the finger for an instant, and then smell your finger. This will tell its own story. In drilling into one of the fillings condensed with the electric mallet more than one patient has exclaimed, "My! Excuse me, but there is something rotten in Denmark." If such fillings had a specific gravity of 17 or 18, the different layers of gold would not be filled with the products of fermentation. This process goes on between the different layers of gold which have not been properly condensed. Later on it will be discovered that these ill-smelling fillings are filled with micro-organisms which materially interfere with the health of the individual in whose teeth such corruption exists.

It is not necessary to enlarge upon these four facts. There remains, then, but one thing to discuss, and that is the doctor's expressed views regarding the uselessness of definite knowledge. I am very sorry that Dr. Rhein said what he did about my statement of the size of the cavity in the lower left first bicuspid. The size of the cavity in two directions, the size of the gingival seat, as well as the size of the first three pieces of gold used, were all given. This was done for the reason that anybody who was interested and who had a Boley gauge, could make a similar operation and thus ascertain for himself whether or not the contention which I had made was correct. I try to be definite and to so place matters before the members of the profession that others can prove my results, provided there is any question regarding them. I must leave for others to judge whether or not this is the right way of doing things. In all kindness, however, I feel that it is preferable to go into detail and be exact, than to place into print under my name, "A cavity, a distal approximating occlusal cavity, in a molar." Over two-thirds of the discussions which take place on all topics are on account of men not being exact in their statements. If I am to judge from the contents of seven different dental journals which come to me each month, I can readily say that there is a constant increase in the ranks of those who are using more exact terms and who are following more definite methods. It is to be deplored that any man should be willing to go into print and acknowledge that he was opposed to definite knowledge. The use of the Boley gauge and a dentometer will increase as the value of definite knowledge is more greatly appreciated, Dr. Rhein's unfortunate remarks to the contrary notwithstanding.

I have a certain kind of respect for a man who will discuss scientific subjects from a scientific basis. I have too little time at my disposal to waste it in being personal and in ridiculing what I know nothing about. If I could not be foremost in assisting others to obtain definite knowledge, I should at least do the best that I could for them. And if I could not help others to raise the standard to a higher plane, I should not allow my name to stand at the head of an answer of the kind Dr. Rhein has sent to my discussion of his ideas.

(On account of the limited time at my disposal, I have been compelled to dictate this answer in greatest haste and at different times, therefore every apology is offered for the same.)

